# The prevalence of chronic dehydration and associated with cardiometabolic risks among agriculture and aquaculture workers

**DOI:** 10.3389/fpubh.2023.1183557

**Published:** 2023-09-07

**Authors:** Ta-Chin Wang, Yuan-Hsiung Tsai, Jen-Tsung Yang, Ming-Shyang Lin, Yu-Chih Lin, Tung-Jung Huang, Mei-Yen Chen

**Affiliations:** ^1^Department of Neurosurgery, Chang Gung Memorial Hospital, Chiayi, Taiwan; ^2^Department of Radiology, Chang Gung Memorial Hospital, Chiayi, Taiwan; ^3^Department of Cardiology, Chang Gung Memorial Hospital, Chiayi, Taiwan; ^4^Department of Family Medicine, Chang Gung Memorial Hospital, Yunlin, Taiwan; ^5^Department of Pulmonary and Critical Care, Chang Gung Memorial Hospital, Yunlin, Taiwan; ^6^Department of Nursing, Chang Gung University of Science and Technology, Chiayi, Taiwan; ^7^School of Nursing, Chang Gung University, Taoyuan, Taiwan

**Keywords:** dehydration, serum osmolality, cardiometabolic risks, agriculture, aquaculture

## Abstract

**Background:**

Chronic dehydration is associated with complications and mortality in acute ischemic stroke patients. Prior literature indicates that farmers and fishery workers are commonly affected by cardiometabolic diseases and there is a need for early prevention of stroke. This study explores the prevalence of dehydration and the association of cardiometabolic risk profiles in agricultural and aquaculture workers.

**Methods:**

We conducted a community-based, cross-sectional study of agriculture and aquaculture workers in Yunlin County of Taiwan between August 1 and December 31, 2021. Data on demographic characteristics and health-related lifestyles were collected through one-on-one interviews using a questionnaire. The threshold for dehydration is defined as serum osmolality ≥295 mOsm/kg, and physiological biomarkers were collected from a collaborating hospital. Multivariable logistic regression analyses adjusted for demographic characteristics were performed to investigate the association between dehydration levels, cardiometabolic risks, and health-related behaviors.

**Results:**

A total of 962 Taiwanese agriculture and aquaculture workers who were predominantly women (65%) with a mean age of 64 years (SD = 13.8) were enrolled. The findings showed a high prevalence of dehydration (36%), metabolic syndrome (44.5%), abnormal waist circumference (64.4%), and abnormal blood pressure (68.5%). Multivariate logistic regression demonstrated that dehydration was significantly associated with metabolic syndrome (*p* < 0.001), 10-year stroke risk prediction (*p* < 0.001), and an unhealthy lifestyle (*p* < 0.001).

**Conclusion:**

The prevalence of chronic dehydration was higher in Taiwanese agriculture and aquaculture workers, which was significantly associated with cardiometabolic risks and unhealthy lifestyles.

## 1. Introduction

Cardiovascular diseases (CVDs) are a group of disorders of the heart and blood vessels, such as coronary heart disease, hypertension, and cerebrovascular accident (stroke). Ischemic heart disease and stroke are the two leading causes of death worldwide (1) as well as in Taiwan, where it ranks as the 2nd and 4th most common cause of death among the top ten causes (2). During the past three decades, global stroke incidence, prevalence, mortality rates, and disability duration have increased by 70, 85, 43, and 32%, respectively (3). The burden of stroke not only impacts the independence of the individual or adversely affects the quality of life but also restricts family function (4, 5). Moreover, stroke has gained importance as a huge public health issue in many less developed countries (6).

Risk factors for stroke include hypertension, diabetes, heart diseases, atrial fibrillation, hyperlipidemia, inflammation, infection, smoking, family history, sleep disorders, male, and older age (3, 7). Age, sex, and some genetic issues are non-modifiable risk factors for stroke, whereas hypertension, diabetes, obesity, unhealthy diet (e.g., low consumption of vegetables and fruits), poor oral hygiene, sleep disorders, tobacco use, and physical inactivity are modifiable lifestyle factors (1, 7–10). The pathophysiology of stroke is well characterized, and most cases are attributed to modifiable factors (7, 11, 12). It is important to detect the risk factors of stroke early to enable management with medications and modification of lifestyle behaviors. Based on the natural disease history, most patients with CVDs or diabetes experience a period of metabolic syndrome (MetS), which is estimated to be one-third of the top ten causes of total deaths in Taiwan (13). The components of MetS include elevated: systolic or diastolic blood pressure, fasting blood glucose, triglycerides, low-density lipoprotein cholesterol, and central obesity (13, 14). To provide an understandable prevention strategy, the Taiwanese government used a formula modified by the Framingham risk prediction model and stratified by male and female to predict the 10-year risk of stroke in the Taiwanese adult population (14, 15).

Although dehydration is not listed as a risk factor for stroke, it is commonly found in admitted stroke patients and is associated with poor outcomes such as venous thromboembolism, in acute ischemic stroke patients (16–18). Previous studies have shown that dehydration upon ischemic stroke admission is associated with in-hospital complications, disability, infection, or death. Admission dehydration status may be a significant and independent predictor of short-term mortality in patients with spontaneous intracerebral hemorrhage (17–19). In addition, dehydration on admission is significantly associated with postoperative complication rates, in-hospital mortality, and length of hospital stay (20, 21). Dehydration is common in hospitals and communities of older people (22–25). This might be attributed to the complexity of dehydration assessment which requires a combination of physiological and laboratory processes. In addition, a universally accepted definition of dehydration is lacking (22). Thus, dehydration assessment is not regularly performed and is underutilized in routine care, let alone in community settings for outdoor workers. Recently, the direct measurement of plasma osmolarity (pOsm) has been recommended as a valuable marker for determining dehydration (24, 25). A pOsm >295 or > 300 mOsm/kg has been defined as impending or hyperosmolar dehydration (21, 22, 26, 27). Osmolality indicates the concentration of all particles dissolved in body fluid (28, 29).

Chronic dehydration is primarily due to insufficient fluid intake over a lengthy period to replace obligatory fluid loss and is characterized by increased serum osmolality (24, 26). The risk factors for chronic dehydration include older age, long-term care residents, cognitive impairment, female, and use of diuretics (21, 23, 30). In the county of Yunlin, the proportion of older people (>65 years) ranks 3^rd^ among 22 counties in Taiwan with most people working in farms and fisheries; both occupations were more impacted by cardiometabolic diseases (31, 32). Older adults are prone to developing dehydration with immobility, impaired thirst, and cardiometabolic diseases. There are no previous studies on the prevalence of dehydration among agricultural and aquaculture workers. Therefore, this novel study aimed to explore the prevalence of chronic dehydration and its possible association with cardiometabolic risks and the 10-year risk of stroke.

## 2. Materials and methods

### 2.1. Design and population

This community-based, cross-sectional study was conducted between August and December 2021, in rural Yunlin County, Taiwan. A higher proportion of older people live around the five townships in the western coastal areas, and this county has the 3rd highest population of more than 20% aged >65 years. The research team initiated community health screening and collaborated with the local hospitals and district health centers. The inclusion criteria were (a) age > 20 years, able to communicate in Mandarin or Taiwanese; (b) able to walk to the community activity center; (c) current or former worker in farming or fisheries; and (d) willingness to join this study and submit the informed consent form. The exclusion criteria included (a) inability to answer questions or refusal to provide informed consent; (b) chronic kidney diseases with the estimated glomerular filtration rate (eGFR<60 mL/min/1.73 m^2^); and (c) dialysis or heart failure were excluded from this study.

### 2.2. Procedure and ethical considerations

Before conducting this study, we received approval from the Institutional Review Board of the Ethics Committee of Chang Gung Memorial Hospital (IRB 202002186B0). The village heads had sent messages regarding free health check-ups and invited adult villagers to participate in this study. The research assistants described the study procedures. We collected a blood sample of less than 20 mL from each participant in various communities between 7–8 am, following an overnight fasting period of 8 h. Blood chemistry includes blood glucose, triglycerides, glycosylated hemoglobin, and low-high-density lipoprotein cholesterol. All the participants were notified of the study’s purpose and had the right to decide whether to participate in the program.

### 2.3. Measurement

*Demographic characteristics and anthropometric assessments* included sex, age, educational level, occupation, marital status, living arrangements, and the number of natural teeth. The height (cm) and body weight (kg) were recorded. Waist circumference was measured using a soft tap and was defined at the umbilical level while standing without stress. Body mass index (BMI) was calculated using a standard formula (kg/m^2^). Two blood pressure measurements of each participant were recorded using an automated oscillometric monitor (Omega 1,400; Florida, United States) in the sitting position after 5 min of rest.

*Health-related lifestyle* included the following seven behaviors, which were based on previous studies related to health promotion lifestyle (8, 10, 33): (1) intake of at least three servings (≥ 1.5 bowls) of vegetables/per day, (2) consumption of two servings (one bowl) of fruits per day, (3) at least 1,500 mL of plain water intake per day, (4) regular exercise for 30 min ≥ 3 times or 150 min/week of physical activity. Responses were recorded as low (never/seldom) or high (usually/always), (5) smoking cigarettes, or (6) chewing betel nuts, (never or current/former use), and (7) the presence of sleep distress indicated by difficulty in falling asleep currently or during the past week. Responses included 0 = never, 1 = slightly, 2 = ordinarily, 3 = quite often, and 4 = utmost.

*Dehydration* was measured by plasma osmolarity and pOsm ≥295 or ≥ 300 (mOsm/kg) was classified as impending or hyperosmolar dehydration (21, 26, 27). For every participant, blood samples were gathered in the early morning, specifically between 7 and 8 am, prior to their breakfast, following a fasting period of at least 8 h. This fasting interval only permitted water intake. Furthermore, water consumption was constrained within the 30 min preceding the blood test. An osmometer (Model 3,250; Advanced Instruments, Massachusetts, United States) was used to measure serum osmolality using the freezing point depression method.

*Cardiometabolic risks* were measured based on the following five abnormal biomarkers (14): (1) central obesity: elevated waist circumference (WC) in males/females ≥90/80 cm, (2) elevated systolic/diastolic blood pressure (SBP/DBP): SBP ≥130 mmHg, DBP ≥85 mmHg, (3) glycosylated hemoglobin (HbA1c) ≥ 5.6%, (4) elevated fasting triglyceride (TG) ≥150 mg/dL, and (5) low-high-density lipoprotein cholesterol (HDL-C) <40/50 mg/dL for male and female. Participants currently using medications for hypertension, hyperlipidemia, or diabetes were categorized as abnormal biomarkers. Individuals with ≥3 risk factors were defined as having MetS (13).

The *risk of stroke* was calculated using the Framingham equation model to predict 10 years of risk for stroke probability (14), which was stratified by male and female. The parameters for women included six variables: age, SBP, WC, history of hypertension and diabetes, and smoking habits. The parameters for men included four variables: age, SBP, FBG, and TG. The Framingham score was classified as low risk (<10%), medium 10–20%, and high risk (>20%) (14, 15).

### 2.4. Statistical analyses

According to serum osmolality, the dehydration status of the participants was classified into an ordinal variable with three levels: normal (<295 mOsm/kg), impending (295–299 mOsm/kg), and hyperosmolar (≥300 mOsm/kg). The linear trend of the demographics and characteristics over the ordinal dehydration status was tested using the linear contrast of one-way analysis of variance for continuous variables or Cochran–Armitage analysis for categorical variables. The association between each health-related lifestyle (including vegetable, fruit, and water intake, exercise, substance use, oral hygiene, and sleep disorders) and the three levels of dehydration status was evaluated using the proportional odds model. However, a binary logistic regression model assessed the correlation between ordinal dehydration status and cardiometabolic risk factors. Finally, we investigated the relationship between dehydration status, the number of MetS components, and 10-year stroke risk using a proportional odds model. Each regression model was adjusted for sex, age, and education level. All tests were 2-tailed and *p* < 0.05 was considered statistically significant. Data analyses were conducted using SPSS version 26 (IBM SPSS Inc., Chicago, Illinois, United States).

## 3. Results

### 3.1. Demographics and characteristics of the participants

**Figure 1:**
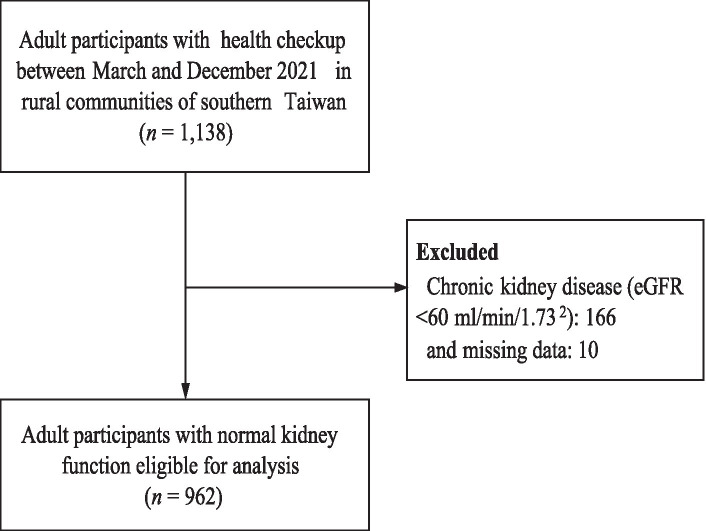
Flow chart of participants’ recruitment.

A total of 1,138 current or former agricultural or aquaculture adult workers were enrolled in this study. We excluded 166 participants with chronic kidney disease (estimated glomerular filtration rate < 60 mL/min/1.73^2^), dialysis, heart failure, and 10 with incomplete data, yielding 962 participants who were eligible for analysis (Figure 1). Table 1 shows that the mean age was 64 years [standard deviation (SD) = 13.8 years] and approximately 65% of the participants were women. The education level was low, with a mean of 7 years (SD = 5.5 years). Noticeably, nearly half of the participants (44.5%) had MetS, and more than half (61.9%) had a high risk of a 10-years stroke. Thirty-six percent of the participants were categorized with a dehydration status of which 26% had impending dehydration and 10% had hyperosmolar dehydration. The results indicated that participants with more severe dehydration status tended to be older, had a lower education level, had inadequate consumption of vegetables and fruits, were less likely to adopt regular exercise, chewed betel nuts, had fewer real teeth, had more sleep disorders and cardiometabolic risk factors, and had a greater risk of 10-years stroke prediction (*p* for trend <0.05).

**Table 1:** Demographics and characteristics of the participants by different dehydration status.

Variable	Total (*n* = 962)	Dehydration status			*p* for trend	Normal (*n* = 615, 64%)	Impending (*n* = 255, 26%)	Hyperosmolar (*n* = 92, 10%)
Demographics					
Age, year	64.0 ± 13.8	61.9 ± 14.5	68.0 ± 11.6	67.1 ± 11.4	<0.001
Male	340 (35.3)	213 (34.6)	91 (35.7)	36 (39.1)	0.696
Education level, year	7.0 ± 5.5	8.0 ± 5.5	5.4 ± 5.1	4.7 ± 4.8	<0.001
Married	680 (70.7)	438 (71.2)	177 (69.4)	65 (70.7)	0.867
Live alone	123 (12.8)	71 (11.5)	38 (14.9)	14 (15.2)	0.307
Dietary habit					
Intake vegetable ≥3 portions per day	683 (71.0)	459 (74.6)	171 (67.1)	53 (57.6)	0.001
Intake fruit ≥2 portions per day	614 (63.8)	418 (68.0)	153 (60.0)	43 (46.7)	<0.001
Intake of water ≥1,500 cc per day	586 (60.9)	377 (61.3)	152 (59.6)	57 (62.0)	0.877
Regular exercise	397 (41.3)	266 (43.3)	105 (41.2)	26 (28.3)	0.024
Substance use					
Smoking	107 (11.1)	65 (10.6)	31 (12.2)	11 (12.0)	0.767
Betel nut	45 (4.7)	24 (3.9)	10 (3.9)	11 (12.0)	0.002
Oral hygiene					
Number of real teeth	18.1 ± 11.2	19.4 ± 11.0	15.8 ± 11.5	16.2 ± 10.9	<0.001
Number of real teeth ≥20	556 (57.8)	392 (63.7)	119 (46.7)	45 (48.9)	<0.001
Sleep disorder	471 (49.0)	283 (46.0)	132 (51.8)	56 (60.9)	0.005
Anthropometry
Height, cm	158.5 ± 8.4	158.8 ± 8.4	158.0 ± 8.3	158.2 ± 8.7	0.437
Weight, kg	63.5 ± 11.5	63.2 ± 11.7	63.8 ± 11.2	64.7 ± 11.7	0.438
Body mass index, kg/m^2^	25.2 ± 3.8	25.0 ± 3.9	25.5 ± 3.8	25.8 ± 3.8	0.063
Data of metabolic syndrome (MetS)					
Waist circumference (WC), cm	86.7 ± 10.4	85.9 ± 10.7	87.8 ± 9.8	88.8 ± 9.4	0.006
Diastolic blood pressure, mmHg	77.6 ± 12.5	76.9 ± 12.1	78.9 ± 12.9	79.2 ± 13.9	0.046
Systolic blood pressure, mmHg	134.2 ± 21.9	132.0 ± 21.3	138.2 ± 23.3	137.5 ± 20.0	<0.001
Glycated hemoglobin, %	6.1 ± 1.0	5.9 ± 0.8	6.2 ± 0.9	7.0 ± 1.6	<0.001
Triglyceride, mg/dL	128.6 ± 84.0	122.2 ± 74.8	131.8 ± 91.9	162.3 ± 108.2	<0.001
High-density lipoprotein, mg/dL	54.5 ± 14.1	55.5 ± 14.4	54.0 ± 13.5	49.6 ± 13.3	0.001
Abnormal of cardiometabolic risks					
Central obesity (WC)^a^	620 (64.4)	371 (60.3)	185 (72.5)	64 (69.6)	0.002
Blood pressure^b^	659 (68.5)	397 (64.6)	187 (73.3)	75 (81.5)	0.001
Glycated hemoglobin^c^	387 (40.2)	204 (33.2)	123 (48.2)	60 (65.2)	<0.001
Triglyceride^d^	269 (28.0)	161 (26.2)	69 (27.1)	39 (42.4)	0.005
High-density lipoprotein^e^	255 (26.5)	150 (24.4)	68 (26.7)	37 (40.2)	0.006
Metabolic syndrome (MetS)^f^	428 (44.5)	240 (39.0)	128 (50.2)	60 (65.2)	<0.001
Number of components of MetS	2.3 ± 1.3	2.1 ± 1.3	2.5 ± 1.3	3.0 ± 1.3	<0.001
10-years stroke risk					<0.001
Low risk	192 (20.0)	154 (25.0)	32 (12.5)	6 (6.5)	
Moderate risk	175 (18.2)	122 (19.8)	37 (14.5)	16 (17.4)	
High risk	595 (61.9)	339 (55.1)	186 (72.9)	70 (76.1)	

Data were presented as frequency (percentage) or mean ± standard deviation.

a WC, waist circumference, male > 90 cm, female > 80 cm.

b Blood pressure > 130/85 mmHg; systolic blood pressure / diastolic blood pressure.

c Glycated hemoglobin ≥ 5.6%.

d Triglyceride ≥ 150 mg/dL.

e HDL, high-density lipoprotein, male < 40 mg/dL, female < 50 mg/dL.

f Number of components of MetS ≥ 3 components.

### 3.2. Association between health-related lifestyle and dehydration status

After adjusting for age, sex, and education level, the results showed that a sufficient intake of vegetables (odds ratio [OR] 0.67, 95% confidence interval [CI] 0.50–0.89) and fruits (OR 0.65, 95% CI 0.50–0.86) were significantly associated with a lower level of dehydration status (Table 2). In contrast, betel nut chewing (OR 2.09, 95% CI 1.12–3.91) and the presence of sleep disorder (OR 1.36, 95% CI 1.04–1.78) were significantly associated with a higher level of dehydration status. In addition, adopting regular exercise was borderline significantly associated with a lower level of dehydration (OR 0.76, 95% CI 0.58–1.00; *p* = 0.053).

**Table 2:** Association between health-related lifestyle and dehydration status (an ordinal variable with 3 levels).

Predictor	Unadjusted		Adjusted^*^	
	Odds ratio (95% CI)	*p* value	Odds ratio (95% CI)	*p* value
Dietary habit				
Intake vegetable ≥3 portions per day	0.60 (0.46–0.79)	<0.001	0.67 (0.50–0.89)	0.005
Intake fruit ≥2 portions per day	0.59 (0.45–0.77)	<0.001	0.65 (0.50–0.86)	0.002
Intake of water ≥1,500 cc per day	0.97 (0.74–1.26)	0.808	1.09 (0.82–1.43)	0.561
Regular exercise	0.76 (0.58–0.99)	0.041	0.76 (0.58–1.00)	0.053
Substance use				
Smoking	1.15 (0.77–1.73)	0.483	1.19 (0.75–1.87)	0.458
Betel nut	1.94 (1.07–3.53)	0.029	2.09 (1.12–3.91)	0.020
Oral hygiene				
Number of real teeth	0.97 (0.96–0.99)	<0.001	0.999 (0.98–1.01)	0.843
Number of real teeth ≥20	0.53 (0.41–0.69)	<0.001	0.86 (0.64–1.17)	0.338
Sleep disorder	1.42 (1.10–1.85)	0.007	1.36 (1.04–1.78)	0.025

*Adjusted by age, sex, and education level.

### 3.3. Association between dehydration and cardiometabolic and stroke risk

Table 3 shows that a higher level of dehydration status was significantly associated with a greater risk of cardiometabolic disorders, including abnormal glycated hemoglobin (OR 1.67, 95% CI 1.36–2.06), abnormal triglyceride (OR 1.28, 95% CI 1.03–1.58), abnormal high-density lipoprotein (OR 1.30, 95% CI 1.05–1.61), and MetS (OR 1.46, 95% CI 1.19–1.79). Additionally, a higher level of dehydration status was associated with a greater number of MetS components (odds ratio [OR] 1.49, 95% CI 1.25–1.78). Furthermore, a higher level of dehydration status was significantly associated with a greater 10-year stroke risk prediction (OR 1.37, 95% CI 1.06–1.78).

**Table 3:** Association between dehydration status (an ordinal variable with 3 levels) and cardiometabolic risk factors.

Outcome	Unadjusted		Adjusted^*^	
	Odds ratio (95% CI)	*p* for trend	Odds ratio (95% CI)	*p* for trend
Abnormal of cardiometabolic risk factors				
Central obesity (WC)^a^	1.38 (1.12–1.71)	0.002	1.17 (0.94–1.46)	0.170
Blood pressure^b^	1.54 (1.23–1.92)	<0.001	1.26 (1.00–1.60)	0.052
Glycated hemoglobin^c^	1.92 (1.57–2.34)	<0.001	1.67 (1.36–2.06)	<0.001
Triglyceride^d^	1.32 (1.07–1.62)	0.009	1.28 (1.03–1.58)	0.024
High-density lipoprotein^e^	1.35 (1.09–1.66)	0.005	1.30 (1.05–1.61)	0.018
Metabolic syndrome (MetS)^f^	1.66 (1.37–2.02)	<0.001	1.46 (1.19–1.79)	<0.001
Number of components of MetS	1.78 (1.49–2.12)	<0.001	1.49 (1.25–1.78)	<0.001
10-years stroke risk (an ordinal variable with 3 levels)	1.87 (1.51–2.32)	<0.001	1.37 (1.06–1.78)	0.018

*Adjusted by age, sex, and education level.

a WC, waist circumference, male > 90 cm, female > 80 cm.

b Blood pressure > 130/85 mmHg; systolic blood pressure/diastolic blood pressure.

c Glycated hemoglobin ≥ 5.6%.

d Triglyceride ≥ 150 mg/dL.

e HDL, high-density lipoprotein, male < 40 mg/dL, female < 50 mg/dL.

f MetS ≥ 3 abnormal cardiometabolic risk factors.

## 4. Discussion

This study has three important findings. First, a high prevalence of chronic dehydration and cardiometabolic risk was observed. Second, chronic dehydration was significantly associated with metabolic syndrome and the risk of 10-year stroke prediction. Third, chronic dehydration was significantly associated with an unhealthy lifestyle, such as inadequate adoption of vegetables, fruits, and exercise, as well as increased betel nut chewing, and sleep distress. Our study showed that more than one-third of the participants had impending hyperosmolar dehydration. This result was higher than that of previous studies; in Japan, Nagae et al. (24) found that 16.9% of nursing home residents had dehydration; in Italy, Zanetti et al. (21) reported 20.4% of preoperative patients with dehydration; in a systematic review of nursing home residents, Paulis et al. (25) found dehydration rate between 0.8 and 38.5%. This may be attributed to the use of different healthcare settings and methods to classify the dehydration status. Although serum osmolality is recommended the preferred biomarker for determining dehydration (22, 27), however, due to the serum osmolarity changing over time due to water consumption, it is better to perform serial measurements to improve the consistency. Nevertheless, the conventional instruments for assessing dehydration from blood and urine samples are expensive and time-consuming; collecting blood and urine samples is inconvenient and not feasible for community adults in rural areas. Therefore, it is necessary to further investigate the dehydration status of community adults in different situations and seasons. For instance, using simpler and noninvasive methods to detect chronic dehydration, such as a portable miniaturized device for dehydration diagnosis with clinical saliva samples, a time-saving and convenient solution, may serve as a good assessment device in the future (34).

The present study indicated a high prevalence of cardiometabolic risk factors and metabolic syndrome. These findings echo those of a representative previous survey from nationwide samples that both farmers and fishery workers were more affected by cardiometabolic diseases (32). Due to an increasingly aging society, older adult people tend to have a higher risk of stroke morbidity and mortality. There is an urgent need to initiate stroke prevention strategies that target modifiable risk factors, which can be classified into three levels: primordial, primary, and secondary prevention (6, 11, 35). According to the American Heart Association, promoting cardiovascular health should include the components of “Life’s Essential 8,” which include a healthy diet, physical activity, avoidance of nicotine exposure, healthy sleep, adequate body mass index, blood lipids, blood glucose, and blood pressure (33). Our study indicated that more than one-fifth of the participants reported sleep distress with the ordinary and utmost level of difficulty in falling asleep. Many participants did not live up to the standards set by *Life’s Essential 8*. Primordial and primary prevention strategies for stroke for these farmers and fishery workers should be initiated. We suggest that clinicians and primary healthcare providers should initiate precision health-promoting programs, including adequate medicines for individuals with cardiometabolic diseases, weight management through healthy eating and regular exercise, avoidance of substance use, improving sleep distress, assessing dehydration, and tackling it through strategies for all agriculture and aquaculture workers. Hence, health education is imperative to emphasize the significance of maintaining daily adequate hydration, especially for individuals with serum osmolality greater than 295.

The present study showed that 60.9% of the participants reported a water intake of >1,500 mL/day. However, this result did not show a correlation with hydration status. Dehydration is mainly caused by insufficient fluid intake and is characterized by increased serum osmolality (24, 26). The inconsistent phenomenon may be due to inaccurate water intake measurements. For instance, in the present study, the frequency of water intake habits was based on self-reports, leading to inaccurate estimations. Moreover, our questionnaire only gave out two options of either above or below the threshold of 1,500 mL/day for all participants. This differs from guidelines recommended by the European Society for Clinical Nutrition and Metabolism, which recommended a threshold of 1,600 and 2000 mL/per day by women and men, respectively, (36). In addition, Wang et al. (37) reported that daily water intake of >2,500 mL was associated with a lower prevalence of renal stone formation. Our study revealed that 40% of participants did not have adequate water intake. In a study in the UK, Jimoh et al. (26) stated that older adults often choose to reduce their fluid intake to help control incontinence and minimize toilet trips. To accurately measure the amount of fluid intake, the use of smart water monitoring products for future studies has been suggested (38).

Further, the dehydration status was significantly associated with lower education levels. Although there is currently no biological plausibility to explain a direct link between the level of education and osmolarity status, an individual with limited education might have a limited understanding of the significance of adequate water intake and rely solely on thirst sensation as a prompt to rehydrate. To gain deeper insights into the causes of inadequate water intake, it is crucial to conduct further investigations that specifically target adults with lower educational backgrounds. Such studies can aid in developing targeted precision health promotion programs that cater to the specific needs of this population.

## 5. Limitations

This study has some limitations. First, the study was conducted in only one county, and the findings might not be generalizable to other populations. Second, we lacked the exact disease history of cardiometabolic diseases and medications used by each participant, both of which may influence serum osmolality and accurate estimation of dehydration status. Since our primary focus was initially on early detection and primary prevention, it is important to acknowledge that hydration status can be influenced by lifestyle modifications, especially in individuals under diuretic medications. It is worth noting that diuretics have been shown in the literature to increase urine production, potentially resulting in dehydration. Future studies should investigate the disparities in dehydration status between individuals with diuretic medication and those who are not, to better understand the implications and potential differences in hydration levels. Third, according to the cardiometabolic risk criteria set by the Taiwan government, fasting blood glucose was identified as a key component of metabolic syndrome. However, in this study, the collaborating hospital chose to measure HbA1c (≥ 5.6%) instead of fasting blood glucose (≥ 100 mg/dL). This decision was made based on the belief that HbA1c levels could provide a more reliable indication of blood glucose levels. Nevertheless, it is important to note that this choice may have an impact on the comparability of the data obtained in this study with that of other studies. Fourth, it is important to note that our evaluation of the relationship between dehydration and stroke was constrained using an indirect risk calculation method. Employing a longitudinal study design that directly assesses the occurrence of strokes would offer more accurate and conclusive information on this relationship.

## 6. Conclusion

This study revealed a high prevalence of dehydration, cardiometabolic risks, and an unhealthy lifestyle in Taiwanese agriculture and aquaculture workers. Additionally, factors associated with dehydration include metabolic syndrome and unhealthy habits. Further studies are needed to explore the association of these factors with dehydration and raise awareness for adequate intake of water.

## Data availability statement

The original contributions presented in the study are included in the article/supplementary material, further inquiries can be directed to the corresponding author.

## Ethics statement

The studies involving humans were approved by the institutional review board of the Chang Gung Memorial Hospital Foundation. The studies were conducted in accordance with the local legislation and institutional requirements. The participants provided their written informed consent to participate in this study.

## Author contributions

T-CW and Y-HT: data curation, analysis, and writing the original draft. M-YC, J-TY, and T-JH: funding acquisition, methodology, project administration, resources, and supervision. Y-CL and M-SL: investigation. T-CW, Y-HT, M-YC, J-TY, T-JH, Y-CL, and M-SL: writing review and editing and conceptualization. All authors contributed to the article and approved the submitted version.

## Funding

The study was supported by a grant from the Ministry of Science and Technology (MOST-110-2314-B-255-004 -MY3), Chang Gung Memorial Hospital (CORPG6K0193), and Formosa Plastic Group (FCRPF6M0011).

## Conflict of interest

The authors declare that the research was conducted in the absence of any commercial or financial relationships that could be construed as a potential conflict of interest.

## Publisher’s note

All claims expressed in this article are solely those of the authors and do not necessarily represent those of their affiliated organizations, or those of the publisher, the editors and the reviewers. Any product that may be evaluated in this article, or claim that may be made by its manufacturer, is not guaranteed or endorsed by the publisher.
